# Prevalence of dry eye and Meibomian gland dysfunction in Central and South America: a systematic review and meta-analysis

**DOI:** 10.1186/s12886-023-03249-w

**Published:** 2024-01-31

**Authors:** Hongan Chen, Paul McCann, Tiffany Lien, Mengli Xiao, Alison G. Abraham, Darren G. Gregory, Scott G. Hauswirth, Riaz Qureshi, Su-Hsun Liu, Ian J. Saldanha, Tianjing Li

**Affiliations:** 1 Department of Ophthalmology, University of Colorado Anschutz Medical Campus, 1675 Aurora Ct, Aurora, CO F731 USA; 2 Department of Epidemiology, Colorado School of Public Health, Aurora, CO USA; 3 Center for Innovative Design & Analysis, Colorado School of Public Health, Aurora, CO USA; 4 Department of Epidemiology, Johns Hopkins Bloomberg School of Public Health, Baltimore, MD USA

**Keywords:** Dry eye syndrome, Meibomian gland disease, Meta-analysis, Prevalence, Central America, South America

## Abstract

**Background:**

Dry eye is one of the most common ophthalmic conditions and can significantly impact quality of life. Meibomian gland dysfunction (MGD) is a major cause of evaporative dry eye.

We sought to conduct a systematic review and meta-analysis to estimate the prevalence and incidence of dry eye and MGD in Central and South America and to identify factors associated with disease burden.

**Methods:**

Data sources Ovid MEDLINE and Embase.

**Study selection:**

A search conducted on August 16, 2021, identified studies published between January 1, 2010, and August 16, 2021, with no restrictions regarding participant age or language of publication. Case reports, case series, case–control studies, and interventional studies were excluded.

**Data extraction and synthesis:**

The review was based on a protocol registered on PROSPERO (CRD42021256934). Risk of bias was assessed in duplicate using a risk of bias tool designed for the purposes of descriptive epidemiological studies. Data were extracted by one investigator and verified by another for accuracy. Prevalence of dry eye and MGD were grouped based on study participant characteristics.

**Main outcomes and measures:**

Prevalence and incidence of dry eye and MGD in Central and South America. Summary estimates from meta-analysis with 95% confidence intervals (CI).

**Results:**

Fourteen studies (11,594 total participants) were included. The population prevalence of dry eye was 13% (95% CI, 12%-14%) in Brazil and 41% (95% CI, 39%-44%) in Mexico based on one study each. Meta-analyses suggested that dry eye prevalence was 70% among indoor workers (95% CI, 56%-80%;* I*^*2*^, 82%; 3 studies), 71% among students (95% CI, 65%-77%; *I*^*2*^, 92%; 3 studies), and 83% in general ophthalmology clinics (95% CI, 77%-88%; *I*^*2*^, 88%; 2 studies). MGD prevalence ranged from 23% among indoor workers (95% CI, 16%-31%; 1 study) to 68% in general ophthalmology clinics (95% CI, 62%-72%; 1 study). No studies reported incidence of dry eye or MGD.

**Conclusions:**

This systematic review and meta-analysis demonstrated considerable variation in the published prevalence of dry eye and MGD among the general population and subpopulations in Central and South America. Local and subpopulation estimates of dry eye disease burden may be valuable to assist needs assessments and implementation of measures to mitigate the condition.

**Supplementary Information:**

The online version contains supplementary material available at 10.1186/s12886-023-03249-w.

## Background

Dry eye disease (DED) is defined as “a multifactorial disease of the ocular surface characterized by a loss of homeostasis of the tear film, and accompanied by ocular symptoms, in which tear film instability and hyperosmolarity, ocular surface inflammation and damage, and neurosensory abnormalities play etiological roles.” [1] Etiologies of DED are classified as aqueous deficient, evaporative, or mixed. Meibomian gland dysfunction (MGD) is characterized by an alteration of the tear film lipid layer and is a major cause of DED [2]. DED has been shown to have significant social and economic burden and adverse effects on quality of life around the globe [3–8]. We will use the term *dry eye* to encompass the wide range of both symptomatic and clinical presentations of DED.

In 2017, the Tear Film and Ocular Surface Society (TFOS) Dry Eye Workshop (DEWS) II Epidemiology Report estimated the prevalence of dry eye to be 5% to 50% worldwide, depending on the study population and definition and diagnostic methods used [9]. Many of the studies used to determine this estimate studied populations in North America, Europe, and Asia. Recently, our group conducted a systematic review and meta-analysis, estimating dry eye prevalence in the United States to be 8.1% (95% confidence interval [CI] 4.9%-13.1%) [10]. At the time of publication of the TFOS DEWS II Epidemiology Report, there were no population-based studies south of the equator, leaving significant gaps in knowledge about the global epidemiology of dry eye.

Central and South America had a combined estimated population of about 616 million people in 2022 [11]. These regions have different socioeconomic and geo-environmental factors from those of previously studied regions, which may affect the magnitude and impact of the dry eye burden. Characterizing the prevalence and incidence of dry eye in these regions will provide greater insight into the population burden of the condition.

The objectives of the current systematic review and meta-analysis are to estimate the prevalence and incidence of dry eye and MGD in Central and South America and to identify factors associated with disease burden.

## Methods

We adapted the methods from a previously registered protocol (CRD42021256934) [12] and followed the Preferred Reporting Items for Systematic Reviews and Meta-analyses (PRISMA) and Meta-analysis of Observational Studies in Epidemiology (MOOSE) guidelines for reporting [13, 14].

### Eligibility criteria

We considered eligible population-based, clinic-based, and secondary healthcare database studies that reported prevalence or incidence of dry eye or MGD in Central and South American countries. We did not exclude studies based on diagnostic criteria used to define dry eye in the studies. We followed guidance from the TFOS DEWS-II report to categorize case definitions, including: (1) Women’s Health Study criteria (i.e., self-reported physician diagnosis and/or self-reported constant or often symptoms) [15], (2) symptoms when signs were not measured (e.g., measured by the 5-item Dry Eye Questionnaire), (3) clinical signs when symptoms were not measured (e.g., tear breakup time), (4) combination of signs and symptoms (distinct from Women’s Health Study criteria), and (5) MGD (e.g., meibomian gland assessment) [9]. We also considered dry eye and MGD definitions based on relevant Current Procedural Terminology (CPT) and International Classification of Disease (ICD) codes*.*

We excluded case reports, case series, case–control studies, interventional studies, as well as studies reported only as abstracts.

### Search strategies

In collaboration with an information specialist from the University of Colorado Strauss Health Sciences Library, we searched Ovid MEDLINE and Embase for studies published between January 1, 2010 and August 16, 2021, to provide current estimates of dry eye and MGD frequency. We included relevant controlled-vocabulary terms (i.e., medical subject headings in MEDLINE, Emtree terms in Embase) and text words (eTable 1). We hand-searched the reference lists of included studies.

We searched the Cochrane Eyes and Vision US Satellite (CEV@US) database of systematic reviews on March 15, 2023, for systematic reviews tagged with *condition: dry eye* and *review type: epidemiology – prevalence/incidence,*and reviewed available reference lists of relevant systematic reviews [16]. We also searched the World Health Organization site on March 15, 2023, using the keywords *dry eye* and *meibomian gland dysfunction*. We retrieved one systematic review and the World Health Organization World Report on Vision, and reviewed the reference lists [17, 18]. No additional studies were retrieved from any of the reference lists.

### Study selection

At both the title/abstract and full-text stages, each record was independently screened by two investigators using Covidence [19]. Discrepancies were resolved via discussion or with a third investigator as needed.

### Data extraction and risk-of-bias assessment

One investigator extracted all relevant study characteristics, methods, and results from each included study using a data extraction form developed on the web-based platform—Systematic Review Data Repository Plus [20]. An independent investigator verified all extracted data with discrepancies resolved via discussion or with a third investigator as needed. For included studies, two investigators independently assessed risk-of-bias using a published risk of bias tool for the purposes of descriptive epidemiological studies [21]. Discrepancies were resolved via discussion or a third investigator as needed.

### Evaluation of heterogeneity

We summarized study characteristics using evidence tables. We investigated clinical heterogeneity by assessing demographic characteristics (e.g., age, sex, region, type of study population). We assessed methodological heterogeneity by evaluating study designs [21]. We assessed statistical heterogeneity by estimating the amount of between-study variance (*τ*^*2*^) and the contribution of between-study variance to the total variability across studies (*I*^*2*^) [22, 23]. We also generated 95% prediction intervals (PIs) – intervals within which the prevalence of a new study would fall if this study were selected at random from the same population of the studies already included in the meta-analysis.

### Meta-analysis

We used mixed-effects models for meta-analyses of dry eye prevalence. We combined dry eye prevalence from each study using symptomatic disease definitions. To model prevalence, we applied generalized linear mixed-effects models with a logit link using the maximum likelihood approach [24]. We reported the summary prevalence and its 95% CIs and 95% PIs. Our primary analysis focused on indoor workers, students or general ophthalmology clinic-based subpopulations for meta-analysis. We also extracted factors associated with dry eye and MGD from included studies and summarized their associations according to their reported odds ratios across studies. We did not conduct meta-analyses for these associations due to methodological heterogeneity. All statistical analyses were conducted using the *metafor *package version 3.8.1 in R version 4.2.2 [25].

## Results

### Search results

Our original search yielded 11,133 records. We excluded 11,117 records due to ineligible populations. After screening 16 full-text reports, we included 14 studies (eFigure 1 in the Supplement). Two full-text reports were excluded due to the study population consisting of pre-existing dry eye disease and neither reported prevalence of dry eye disease [26, 27].

### Characteristics of included studies

The 14 included studies covered three outcomes: dry eye prevalence (*n* = 13), MGD prevalence (*n* = 2) and computer vision syndrome prevalence (*n* = 2) (Table 1) [28–41]. No studies reported incidence of dry eye or MGD. Characteristics of the study populations varied across the 14 studies: general population-based (*n*= 2) [33, 41], indoor and outdoor worker populations (*n*= 3) [28–30], student populations (*n*= 3) [37, 39, 40], and hospital- and clinic-based populations (*n*= 6) [31, 32, 34–36, 38]. Two of the hospital- and clinic-based studies were from general ophthalmology clinics [35, 36]. Definitions of dry eye varied across studies: symptom questionnaires without signs and/or self-reported diagnosis (*n*= 7) [33, 35, 35, 37, 39–41], symptoms and signs (*n*= 4) [28, 32, 34, 36], and signs alone (*n* = 2) (Table 2) [31, 38]. The two MGD prevalence studies used different diagnostic criteria to define MGD: meibum gland quality [36] and MGD stage based on meibum gland quality and expressibility [28]. The two studies that reported prevalence of computer vision syndrome used different symptom questionnaires to define the dry eye component: the Ocular Surface Disease Index (OSDI) [30] and the 5-item Dry Eye Questionnaire (DEQ-5) [37], respectively.

**Table 1:** Study characteristics

Design details								Characteristics of study population		
**Population category**	**Author, publication ** **year**	**Etiology**	**Country**	**Source population**	**Geographical locations**	**Data source (national, ** **regional, local)**	**Sampling scheme;** **(response rate)**	**Female (%)**	**Age (years)**	**Race / ethnicity (%)**
General population-based	Castro, 2018 [33]	DED	Brazil	Brazilian adults aged ≥ 18 years selected from general urban population, from any labor activities, workplace environment, and socioeconomic status	Brazil—North, Northeast, Central-west, Southeast, South geopolitcal regions	Population-based epidemiological study (national)	Sampling scheme—cluster sampling by geopolitical region, consecutive enrollment; (77.7%)	65.5	Mean: 40.5 SD: 17.1	n.r
	Graue-Hernandez, 2018 [41]	DED	Mexico	People aged ≥ 50 years in 39 municipalities	Tlaxcala, Mexico	Population-based epidemiological study (regional)	Sampling scheme—cluster sampling; (58.5%)	59.6	Mean: 64.7 SD: 10.6	n.r
Working populations	Castellanos-Gonzalez, 2016 [28]	DED / MGD	Mexico	Surgical residents (physicians) of the surgical branches of the Specialty Hospital, Western National Medical Center	Guadalajara, Jalisco, Mexico	Single institution clinic or hospital-based study (local)	Sampling scheme—unspecified; (n.r.)	27	Mean: 27.8 SD: 2.1	n.r
	Sanchez-Valerio, 2020 [30]	CVS	Mexico	Office workers at the Autonomous University of Puebla	Puebla, Mexico	Population-based epidemiological study (local)	Sampling scheme—unspecified; (n.r.)	54.6	Mean: 32.1 SD: 7.8 Range: 18 – 45	n.r
	Hernandez-Llamas, 2020 [29]	DED	Mexico	Office workers at Universidad de Monterrey (UDEM) rectory office and construction workers at a new UDEM campus module	Monterrey, Mexico	Single institution clinic or hospital-based study (local)	Sampling scheme-random sampling; (n.r.)	Construction workers 1.3 Office workers 70.3	Construction workers Mean: 35.12 SD: 10.63 Office workers Mean: 33.03 SD: 9.77	n.r
Student populations	Garza-Leon, 2016 [39]	DED	Mexico	All undergraduate and graduate students officially registered at University of Monterrey for the academic year 2014–2015	Monterrey, Mexico	Single institution clinic or hospital-based study (local)	Other—voluntary response sampling; (95.7%)	59.8	Mean: 21.38 SD: 1.79	n.r
	Cartes, 2021 [37]	CVS, DED	Chile	University students from different universities all over Chile who moved their classes online due to the COVID-19 pandemic	Nationwide, Chile	Population-based epidemiological study (national)	Sampling scheme—unspecified; (27.1%)	65	Mean: 21.1 SD: 2.7	n.r
	Garza-Leon, 2021 [40]	DED	Mexico	High school students from Medical Technical High School at Nuevo León Autonomous University	Monterrey, Mexico	Single institution clinic or hospital-based study (local)	Sampling scheme—non-probabalistic random sampling; (n.r.)	55.7	Mean: 16 SD: 0.96	n.r
Hospital- and clinic-based populations	Skare, 2012 [32]	DED	Brazil	Pregnant patients in prenatal care at the Obstetrics Service and non-pregnant patients who had a gynecological appointment at the same hospital	Curitiba, Brazil	Single institution clinic or hospital-based study (local)	Sampling scheme—unspecified; (n.r.)	100	Pregnant Mean: 28.3 SD: 8.3; Non-pregnant Mean: 27.5 SD: 8.5	n.r
	Martinez JD, 2016 [36]	DED/MGD	Mexico	All new patients age 16 to 85 who presented to the tertiary care outpatient clinic of a referral ophthalmology center (Asociación para Evitar la Ceguera)	Mexico City, Mexico	Single institution clinic or hospital-based study (local)	Sampling scheme -consecutive enrollment; (96.6%)	55	Mean: 45 SD: 16	n.r
	Garza-Leon, 2017 [35]	DED	Mexico	Patients who attended for the first time a public or private ophthalmology high specialty center with doctors who are members of the Mexican Group of Research in Visual Sciences	19 states across Mexico	Multi-institution clinic or hospital-based study (national)	Sampling scheme -consecutive enrollment; (n.r.)	59.4	Mean: 50.0 SD: 17.68	n.r
	da Cruz, 2018 [34]	DED	Brazil	Dermatology and ophthalmology clinics of Universidade do Estado do Pará	Belém, Pará, Brazil	Single institution clinic or hospital-based study (local)	Sampling scheme—unspecified; (n.r.)	48.8	Mean: 47.9 SD: 14.6	n.r
	Surmacz, 2021 [31]	DED	Brazil	Adults who visited dermatology clinics for cosmetic reasons and treatment of superficial mycosis during the period of one year in a single Dermatological tertiary center	Curitiba, Brazil	Single institution clinic or hospital-based study (local)	Sampling scheme—convenience sampling; (n.r.)	67.5	Median: 42 IQR: 28 – 55	Eurodescendant: 74.1; Afrodescendant: 25.9; Asiatic: 1.5
	De Freitas, 2021 [38]	DED	Brazil	Brazilian patients seen at a single university hospital with diabetes mellitus and controls without diabetes. DM patients were recruited from a single Endocrinology outpatient clinic of a university Faculdade Evangélica Mackenzie de Curitiba, PR, Brazil hospital and controls from Dermatology Clinic seeking consultation for cosmetic reasons	Curitiba, Brazil	Single institution clinic or hospital-based study (local)	Sampling scheme—convenience sampling; (n.r.)	Diabetes patients 57.5 Controls 55	Diabetes patients Median 59 IQR: 47.2 – 67.0 Controls Median 57 IQR 46.2 – 67.0	n.r

*CVS* Computer vision syndrome, *DED* Dry eye disease, *IQR* Interquartile range, *n*/*a* Not applicable, *n.r.* Not reported, *SD* Standard deviation

**Table 2:** Prevalence of dry eye and Meibomian gland dysfunction

Population category	Author, publication year	Etiology	Measurement method: component(s) contributing to diagnostic standard(s)	Diagnostic standard(s)	Prevalence denominator	Prevalence numerator	Prevalence (%), 95% confidence interval	Point or period of data collection	Characteristics used to stratify prevalence or report an association	Overall risk of bias
General population-based	Castro, 2018 [33]	DED	Self-reported diagnosis; Symptoms	Self-reported diagnosis^a^	3107	317	10.2, 95%CI* 9.2, 11.3	Point: n.r	¶ Sex, country / region; † Age, sex, medical comorbidities, medication use, other—computer exposure, smoking; ‡ Age, sex, medical comorbidities, medication use, other—computer exposure, smoking	M
				Symptoms (questionnaire)^b^	3107	151	4.9, 95%CI* 4.1, 5.7			
				Self reported diagnosis or symptoms (questionnaire)	3107	398	12.9, 95%CI* 11.7, 14.0			
	Graue-Hernandez, 2018 [41]	DED	Symptoms	Symptoms (questionnaire)^c^	1508	621	41.1, 95%CI 38.6, 43.6	Period: July to September 2013	¶ Dry eye symptom severity; † Sex, dry eye symptom severity, medical comorbidities (hypertension, diabetes mellitus), medication use (hypoglycemic, anti-hypertensive), country or region (rural/urban), other-smoking status, alcohol consumption, wearing glasses, cataract surgery, occupation, level of education; ‡ Sex, dry eye symptom severity, other–smoking index, ever alcohol consumption, medication use (anti-hypertensive)	M
Working populations	Castellanos-Gonzalez, 2016 [28]	DED / MGD	Symptoms; signs	Symptoms (questionnaire)^d^	123	69	56.1, 95%CI* 46.9, 65.0	Point: 2014	¶ Medical comorbidities, medication use; other—year of residency, makeup use, microscope use; † n.r.; ‡ n.r.	M
				TBUT^e^	123	70	56.9, 95%CI* 47.7, 65.8			
				Corneal staining^f^	123	30	24.4, 95%CI* 17.1, 32.9			
				Schirmer I test^g^	123	0	0, 95%CI* 0, 2.9^§^			
				Meibomian gland dysfunction^h^	123	28	22.7, 95%CI* 15.7, 31.2			
	Sanchez-Valerio, 2020 [30]	CVS	Symptoms; signs	Symptoms (questionnaire)^d^	108	86*	79.7, 95%CI* 70.8, 86.7	Point: n.r	¶ Other—computer exposure degree; † Other—computer exposure times; ‡ n.r.	M
				TBUT^e^	108	105*	97.2, 95%CI* 92.1, 99.4			
				Ocular surface staining^i^	108	48*	44.4, 95%CI* 34.8, 54.3			
				Schirmer I test^j^	108	29*	26.9, 95%CI* 18.8, 36.2			
	Hernandez-Llamas, 2020 [29]	DED	Symptoms	Symptoms (questionnaire)^d^	Construction workers 149	Construction workers 53	Construction workers 35.6, 95% CI* 27.9, 43.8	Period: October and December 2017	¶ Age, sex, medical comorbidities (systemic disease), other–smoking status, contact lens use, computer hours/day, working hours/day, ocular disease, gender, smoking status, medical comorbidities, other– contact lens use, smoking status, office vs. construction worker, ocular disease; † Gender, smoking status, medical comorbidities, other– contact lens use, smoking status, office vs. construction worker, ocular disease; ‡ Gender, smoking status, medical comorbidities, other– contact lens use, smoking status, office vs. construction worker, ocular disease	H
					Office workers 155	Office workers 112	Office workers 72.3, 95% CI* 64.5, 79.1			
Student populations	Garza-Leon, 2016 [39]	DED	Symptoms	Symptoms (questionnaire)^d^	823	579	70.4, 95% CI* 67.1, 73.5	Point: 2014	¶ Sex, dry eye symptom severity; † n.r.; ‡ Sex, other-smoking status, hours in front of computer, refractive surgery, eye drops user, contact lens user	M
	Cartes, 2021 [37]	CVS, DED	Self-reported diagnosis; Symptoms	Self-reported diagnosis (questionnaire)^k^	1450	85	5.9, 95%CI* 4.7, 7.2	Point: May 2020	¶ Sex, dry eye symptoms; † Sex, medical comorbidities, medication use, other-smoking, contact lens use, screen exposure time; ‡ Sex, screen exposure time, medical comorbidities (previous dry eye disease diagnosis), medication use (allergy medication)	H
				Symptoms (questionnaire)^c^	1450	1123	77.4, 95%CI* 75.2, 79.6			
	Garza-Leon, 2021 [40]	DED	Symptoms	Symptoms (questionnaire)^d^	759	496	65.3, 95% CI* 61.8, 68.7	Point: n.r	¶ Sex, dry eye symptom severity; † Sex, other-contact lens use, contact lens type, contact lens wear overnight; ‡ Sex, other-contact lens use, contact lens type, contact lens wear overnight	M
Hospital- and clinic-based populations	Skare, 2012 [32]	DED	Symptoms; signs	Dry eye sensation	Pregnant 150	Pregnant 24	Pregnant 16.0, 95%CI* 10.5, 22.9	Point: n.r	¶ n.r.; † Pregnant vs non-pregnant and lacrimal dysfunction vs no lacrimal dysfunction, medication use, others—length of pregnancy, number of pregnancies, number of full-term pregnancies, number of abortions; ‡ n.r.	H
					Non-pregnant 150	Non-pregnant 28	Non-pregnant 18.7, 95%CI* 12.8, 25.8			
				Schirmer's test^l^	Pregnant 150	Pregnant 26	Pregnant 17.3, 95%CI* 11.7, 24.4			
					Non-pregnant 150	Non-pregnant 10	Non-pregnant 6.7, 95%CI* 3.2, 11.9			
	Martinez JD, 2016 [36]	DED, MGD	Symptoms, Signs	Symptoms (questionnaire)^d^	338	264	78.1, 95% CI* 73.3, 82.4	Period: November 2012 to February 2013	¶ Sex, age, dry eye symptom severity (OSDI, DEQ), medical comorbidities (diabetes mellitus, arthritis, thyroid problems, dry mouth, acne, depression), medication use (antihypertensive, antihistamine, diuretic, GI ulcer medication, multivitamins, lubricant eye drops), other–smoking, indoors occupation, exposure to air conditioning, contact lens use, ocular sugery; † Symptom severity (OSDI, DEQ-5), aqueous tear deficiency (Schirmer's), evaporative deficiency (TBUT), meibomian gland disease, corneal staining (Oxford) vs. Sex, age, medical comorbidities (diabetes mellitus, arthritis, thyroid problems, dry mouth, acne, depression), medication use (antihypertensive, antihistamine, diuretic, GI ulcer medication, multivitamins, lubricant eye drops), other–smoking, indoors occupation, exposure to air conditioning, contact lens use, ocular sugery; ‡ n.r.	M
				Symptoms (questionnaire)^c^	338	248	73.4, 95% CI* 68.3, 78.0			
				TBUT^m^	338	319	94.4, 95% CI* 91.4, 96.6			
				Corneal staining^n^	338	37	11.0, 95% CI* 7.8, 14.8			
				Schirmer I test^o^	338	74	21.9, 95% CI* 17.6, 26.7			
				Meibomian gland dysfunction^p^	338	228	67.5, 95% CI* 62.2, 72.4			
	Garza-Leon, 2017 [35]	DED	Symptoms	Symptoms (questionnaire)^d^	2270	1967	86.4, 95% CI* 85.2, 88.0	Period: September to December 2014	¶ Sex, dry eye symptoms severity; † Age, dry eye symptoms severity; ‡ Sex, age, other- referring physician specialty	M
	da Cruz, 2018 [34]	DED	Symptoms; signs	Japanese criteria^q^	Psoriasis patients 43 Controls 86	Psoriasis patients 7 Controls 3	Psoriasis patients 16.3, 95%CI* 6.8, 30.7 Controls 3.5, 95%CI* 0.7, 9.9	Period: October 2013 to August 2014	¶ n.r; † n.r.; ‡ n.r.	H
				Symptoms (questionnaire)^d^	Psoriasis patients 43 Controls 86	Psoriasis patients 17 Controls 16	Psoriasis patients 39.5, 95%CI* 24.9, 55.6 Controls 18.6, 95%CI* 11.0, 28.5			
				TBUT^e^	Psoriasis patients 43 Controls 86	Psoriasis patients 23 Controls 35	Psoriasis patients 53.5, 95%CI* 37.7, 68.8 Controls 40.7, 95%CI* 30.2, 51.8			
				Schirmer I test^r^	Psoriasis patients 43 Controls 86	Psoriasis patients 6 Controls 5	Psoriasis patients 13.9, 95%CI* 5.3, 27.9 Controls 5.8, 95%CI* 1.9, 13.1			
				Rose Bengal staining^s^	Psoriasis patients 43 Controls 86	Psoriasis patients 23 Controls 25	Psoriasis patients 53.5, 95%CI* 37.7, 68.8 Controls 29.1, 95%CI* 19.8, 39.9			
	Surmacz, 2021 [31]	DED	Signs	Schirmer I test^t^	135	60	44.4, 95% CI* 35.9, 53.2	Point: n.r	¶ n.r.; † Altered Schirmer's test vs normal and altered TBUT vs normal, age, sex, race/ethnicity, medical comorbidities, medication use, others—smoking, BMI, body composition, OSDI; ‡ n.r.	H
				TBUT^e^	135	108	80, 95%CI* 72.3, 86.4			
	De Freitas, 2021 [38]	DED	Signs	Schirmer I test^l^	Diabetes patients 120	Diabetes patients 46	Diabetes patients 38.3, 95%CI* 29.6, 47.7	Period: April 2018 to March 2019	¶ Severity of dry eye symptoms † Sex, age, medical comorbidities, medication use (metformin, insulin), Other– women at menopause, smoking status ‡ Age medical comorbidities, medication use (metformin, insulin)	H
					Controls 120	Controls 30	Controls 25, 95%CI* 17.6, 33.7			

*CI* Confidence interval; *H* High, *MGD* Meibomian gland dysfunction, *M* Moderate, *n.r.* Not reported, *SD* standard deviation, ¶ Stratified by characteristic, † Characteristic included in a univariate analysis, ‡ Characteristic included in a multivariate analysis, * derived by binomial "exact" calculation (https://sample-size.net/confidence-interval-proportion/); § one-sided 97.5% confidence interval, a – 'Yes' answer to the question “Have you ever been diagnosed (by a clinician) as having dry eye disease?”, b – Womens Health Study questionnaire: Evaluated using the following questions: “How often do your eyes feel dry? and “How often do your eyes feel irritated?”. Response options included “never”, “sometimes”, "often”, and “constantly”. Dry eye defined as the presence of severe symptoms, indicated by "constantly" or "often" response to BOTH questions, c – DEQ-5 score: 7–12 indicates mild to moderate symptoms of DE; > 12 indicates severe symptoms of DE, d – OSDI scale from 0 to 100: 0–12 are considered normal, 13–22 light dry eye symptoms, 23–32 moderate, and > 32 severe symptoms, e – TBUT ≤ 10 s, f – Oxford Schema fluorescein grade ≥ 1, g – Schirmer's I test with anaesthesia < 10 mm, h – Meibomian gland dysfunction stage 2 (Meibomian gland secretion, or “meibum”, quality was assessed in each of the 8 glands of the central third of the lower lid, on a 0–3 scale for each gland: 0 = clear meibum; 1 = cloudy meibum; 2 = cloudy with debris (granular); 3 = thick, like toothpaste. The expressibility of the meibum was assessed from 5 glands: 0 = all glands were expressible; 1 = 3–4 glands were expressible; 2 = 1–2 glands were expressible; 3 = no glands were expressible. Gland dysfunction was classified according to the score obtained: Stage 1 = minimally altered secretions: grade > 2 – < 4; expressiveness: 1. Stage 2 = moderately altered secretions: grade > 4 – < 8; expressiveness: 1. Stage 3 = moderately altered secretions: grade > 8 – < 13; expressiveness: 2. Stage 4 = severely altered secretions: grade > 13; expressiveness: 3), i – Ocular surface damage (fluorescein and Lissamine green) grade ≥ 1, j – Schirmer I test with anesthesia ≤ 5 mm, k – VDT-related symptoms included: soreness, itchiness, pain, dryness, foreign body sensation, redness, visual fatigue, and blurry vision. The frequencies of these symptoms were categorized using a frequency scale in five categories: never, rarely, sometimes, often, and always, and they were graded from 1 (never) to 5 (always) in relation to each answer, l – Schirmer I test without anesthesia ≤ 10 mm, m – TBUT ≤ 5 s, n – Oxford schema corneal fluorescein grade ≥ 2, o – Schirmer I test without anesthesia ≤ 5 mm, p – Meibum gland quality ≥ 2, q – Dry eyes were diagnosed according to Japanese criteria, which required that patients had clinical symptoms and at least two positive results from among the Schirmer I test, the TBUT test, the rose bengal test, and the presence of keratitis. Patients who met only two criteria were classified as having probable dry eyes, r – Schirmer I test without anesthesia ≤ 10 mm / 5 min, s – Rose bengal test, scored from 0–9, considered abnormal if value > 3, t – Schirmer I test without anesthesia ≤ 5 mm in ≥ 1 eye

### Risk-of-bias assessment

Summaries of risk-of-bias assessments for the studies are presented in eTable 2. In total eight studies were deemed moderate risk of bias [28, 30, 33, 35, 36, 39–41] and six studies deemed high risk of bias [29, 31, 32, 34, 37, 38]. None of the studies were determined to be representative of the national population. Further, studies at moderate to high risk of bias were not representative of the target population as defined by the primary studies. Studies with bias introduced by sampling strategies and response rates were also judged to be at moderate to high risk of bias.

### Prevalence of dry eye

Although included in the systematic review, we excluded from meta-analysis cohorts defined by medical conditions (i.e., diabetes, pregnancy, psoriasis) and their controls to focus on the burden of dry eye in general ophthalmology clinics. Of the worker populations in the included studies, all were indoor occupations except for one study of outdoor construction workers. Due to the qualitative difference in working environments, we excluded the outdoor working cohort from meta-analyses. We conducted an exploratory meta-analysis of subpopulations exposed to sustained computer use either for work or for school.

**Fig.1:**
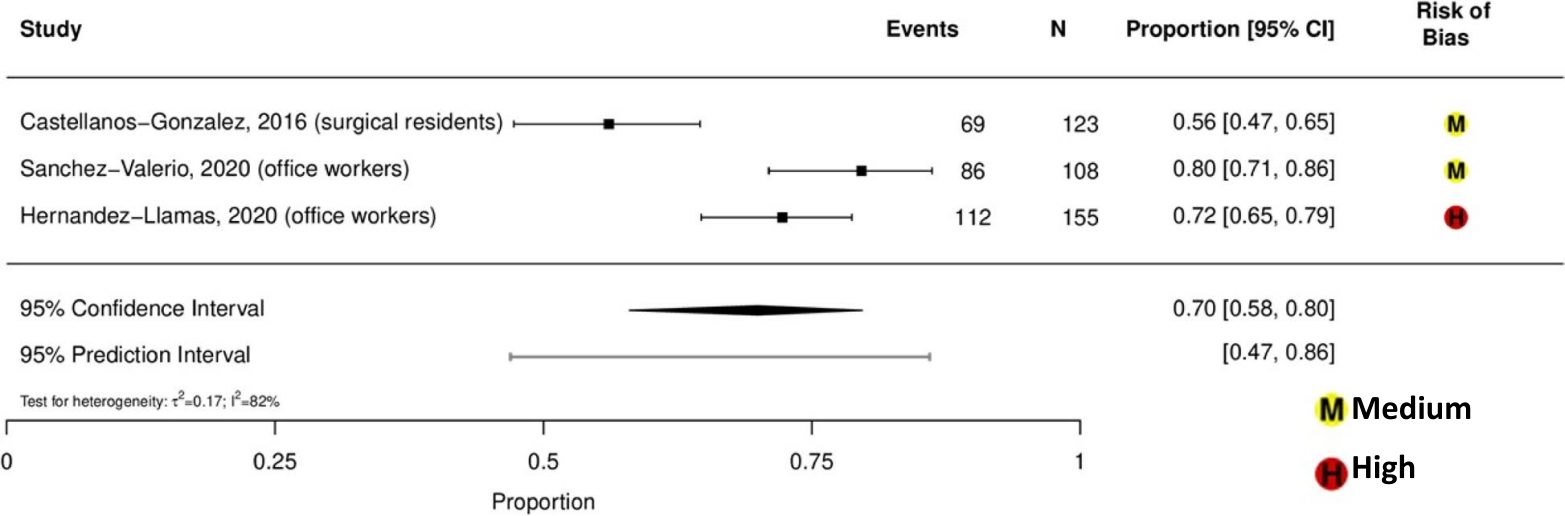
Meta-analysis of the prevalence of dry eye among indoor working populations

**Fig. 2:**
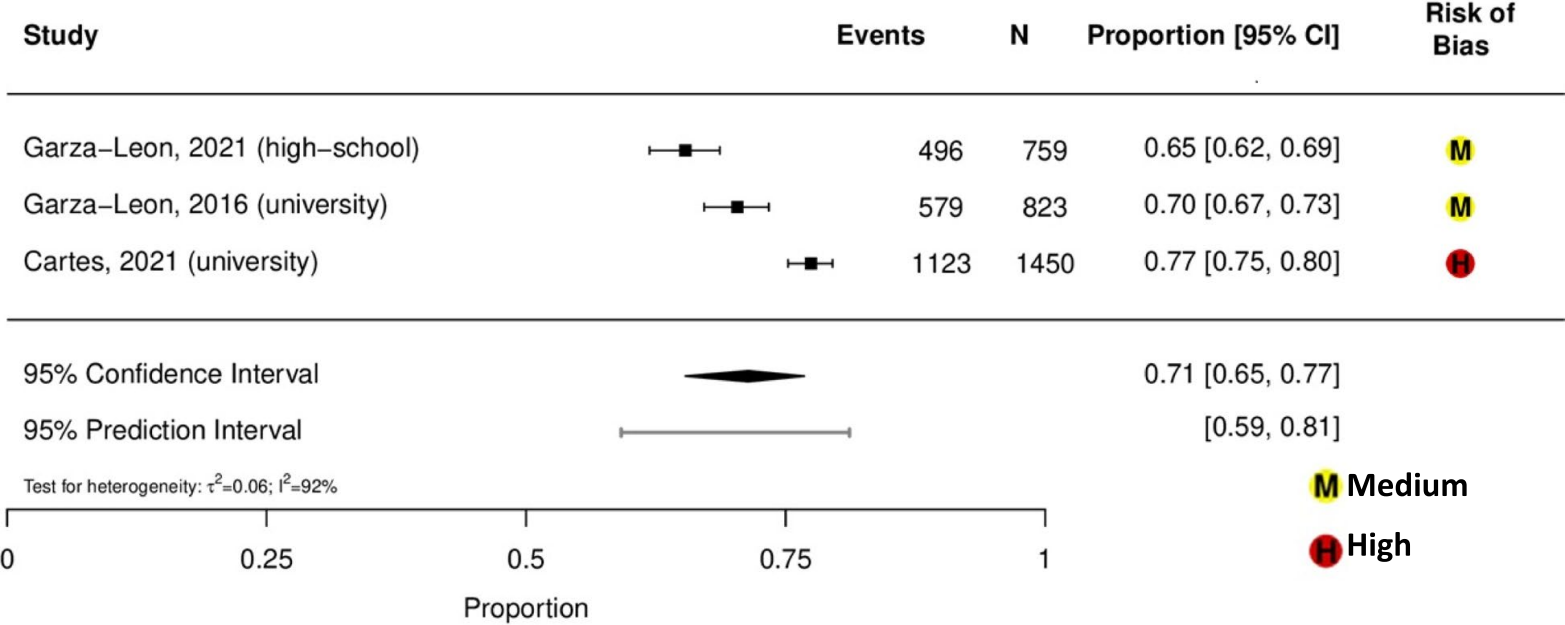
Meta-analysis of the prevalence of dry eye among student populations

**Fig. 3:**
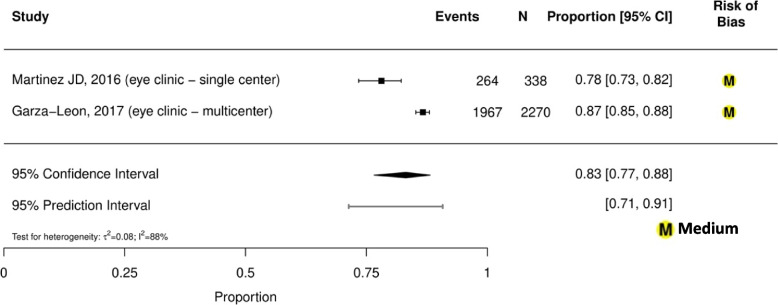
Meta-analysis of the prevalence of dry eye among general ophthalmology clinic-based populations

Prevalence estimates of dry eye among general population-based studies were highly variable, ranging from 13% (95% CI, 12%-14%) in Brazil (aged ≥ 18 years old) to 41% (95% CI, 39%-44%) in Mexico (aged ≥ 50 years old) (eFigure 2) [33, 41]. Prevalence of dry eye among indoor working populations ranged from 56 to 80%, with a summary estimate of 70% (95% CI, 58%-80%; *τ*^2^ = 0.17; *I*^2^ = 82%; 95% PI, 47%-86%; 3 studies, 386 participants; Fig. 1) [28–30]. Prevalence of dry eye among student populations ranged from 65 to 77%, with a summary estimate of 71% (95% CI, 65%-77%; *τ*^2^ = 0.06; *I*^2^ = 92%; 95% PI, 59%-81%; 3 studies, 3,032 participants; Fig. 2) [37, 39, 40]. Prevalence of dry eye among general ophthalmology clinic-based populations ranged from 78 to 87%, with a summary estimate of 83% (95% CI, 77%-88%; *τ*^2^ = 0.08; *I*^2^ = 88%; 95% PI, 71%-91%; 2 studies, 2,608 participants; Fig. 3) [35, 36]. Prevalence of dry eye among student and indoor working populations exposed to sustained computer use ranged from 72 to 80%, with a summary estimate of 77% (95% CI, 75%-79%; *τ*^2^ = 0.00; *I*^2^ = 0%, 95% PI, 75%-79%; 3 studies, 386 participants; eFigure 3).

Summaries of the associations with sex, age, ocular and medical comorbidities, and medication use are outlined in eTables 3–5 in the Supplement. Prevalence of dry eye appears to be associated with increased age (odds ratio [OR] range 1.04–2.02) [31, 33, 35], female sex (OR range 1.49–3.82) [29, 31, 33, 35, 37, 39, 40], contact lens use (OR range 1.12–4.67) [29, 33, 37, 39, 40], and smoking (OR range 1.24–1.44) [33, 37, 39]. While generally associated with increased dry eye prevalence, there is some inconsistent evidence regarding the direction of association with computer use (OR range 0.82–1.49) [30, 33, 37, 39]. In terms of medical comorbidities, one study reported dry eye association with menopause (OR 1.92 [CI 1.37–2.68]), connective tissue disorder (OR 1.93 [CI 1.23–3.02]) and cancer treatment (OR 3.59 [CI 1.71–7.55]) [33].

### Prevalence of MGD and associations

Two studies reported MGD prevalence, which was 68% among general tertiary eye clinic patients and 23% among surgical residents [28, 36]. We observed in both studies that dry eye prevalence assessed by the OSDI was higher than MGD prevalence. One study (338 participants) reported risk factors associated with MGD including older age (per year, OR 1.07, 95% CI 1.05–1.09), male sex (OR 1.7, 95% CI 1.04–2.6), arthritis (OR 7.7, 95% CI 1.001–59), and anti-hypertensive use (OR 2.7, 95% CI 1.3–5.7) [36].

## Discussion

This systematic review and meta-analysis of Central and South American studies published since 2010 estimates the prevalence of dry eye to range from 13% (95% CI, 12%-14%) in Brazil to 41% (95% CI, 39%-44%) in Mexico. Considerable statistical heterogeneity prevented meaningful pooling of their results in meta-analysis. Sources of clinical and methodological heterogeneity between these studies may be attributed to different diagnostic criteria, different eligible age ranges, and different geographic regions. However, a recent systematic review and meta-analysis decided to combine these results using a Bayesian approach and provided a pooled prevalence estimate for South America of 14.7% [18]. In addition, a study published in 2022 reported that the overall prevalence of dry eye was 24.4% in adults from Sao Paulo, Brazil, using the Women’s Health Study criteria [42]. Our meta-analyses of Central and South American populations showed pooled subpopulation estimates of dry eye prevalence ranging from 70% (95% CI, 58%-80%) among indoor working populations [28–30], to 71% (95% CI, 65%-77%) in student populations [37, 39, 40], and 83% (95% CI, 77%-88%) in general ophthalmology clinic-based populations [35, 36]. MGD prevalence ranged from 23% among indoor workers and 68% among general tertiary eye clinic patients [28, 36]. We found no studies of the incidence of dry eye or MGD in these regions.

Interestingly, dry eye was highly prevalent among younger populations, such as high-school and university students, which is consistent with recent dry eye prevalence estimates in Caribbean (84%) and Spanish (51%) university students [43, 44]. Furthermore, a study published in 2021 reported that the overall prevalence of dry eye was 59.6% in undergraduate and medical students from Sao Paulo, Brazil, using OSDI [45]. These additional studies taken together with our meta-analysis, which included studies from three countries in Central and South America, comprise only a small body of evidence. It is worth noting that none our included studies for this subpopulation evaluated local environmental factors, while only one study [37] included medical history in the participant questionnaire. Therefore, we recommend cautious interpretation of these high prevalence estimates and suggest that they warrant further investigation in these and other regions. Nevertheless, we surmise that such high prevalence estimates of dry eye among student populations may stem from prolonged computer or other digital device use and contact lens wear. Given increased screen time since the COVID-19 pandemic, it is possible dry eye prevalence among this population may continue to increase [46–48]. Indeed, our meta-analysis of student and working populations in Central and South America exposed to sustained computer use showed that these subgroups have relatively high prevalence of dry eye [28–30, 37, 39, 40]. Furthermore, most studies that reported an association between computer use and dry eye showed higher dry eye prevalence with more computer use, although one study reported an inverse relationship between computer use and dry eye [39]. However, these associations are limited by cross-sectional designs of the primary studies and causality, reverse or otherwise, cannot be determined.

MGD was reported as less prevalent than dry eye in one eye clinic-based population and one working population [28, 36]. However, without individual participant data, we were unable to determine if MGD represented a subset of dry eye in these populations or if some cases of MGD were asymptomatic.

Risk-of-bias, generalizability, and heterogeneity require consideration. The clinical and methodological heterogeneity across studies corresponds to the wide range of reported prevalence estimates for dry eye, even within well-characterized subpopulations. Several studies used multiple diagnostic criteria to estimate dry eye prevalence [36, 37]. Within each of these studies the reported estimates varied by the diagnostic criteria. We noted poor correlation between prevalence as estimated by patient reported symptom questionnaire cutoff values compared to self-reported diagnosis, suggesting considerable clinical under-ascertainment of disease among university students [37]. For the purposes of meta-analysis, we selected the result provided by patient reported symptom questionnaire cutoff values rather than self-reported diagnosis in order to minimize methodological heterogeneity. Given these findings, and the established poor correlation between subjective and objective measures of DED [49, 50], a set of working diagnostic criteria for DED is necessary for standardization across dry eye epidemiological studies. Our attempts to address between-study heterogeneity in subgroup analysis by population characteristics did not successfully reduce statistical heterogeneity. The remaining residual heterogeneity may be associated with differential exposure to dry eye risk factors within each subpopulation, such as duration of computer use [37, 39, 40], and the variety of occupational exposures [28–30].

We noted that Mexican populations working in indoor environments may have higher prevalence of dry eye compared with Mexican construction workers [29]. However, these results were taken from a single study. Elsewhere, dry eye prevalence was reported to be 51% among Chinese coal workers, and duration and levels of dust exposure were associated with dry eye [51]. We were unable to determine the levels of occupational dust exposure in Mexican construction workers in which dry eye prevalence was 36%. Local climate-related factors (e.g., humidity, temperature, ultraviolet light), pollution, altitude, duration of working shifts, and occupational protection practices may all influence differences in dry eye prevalence among these working populations [48]. Also, the highly localized source populations and small sample sizes included in our systematic review may limit the generalizability and comparability with other populations. Overall, there is some evidence that dry eye is highly prevalent in younger and older South American adults and occupational exposures may have an impact.

The prevalence of dry eye was estimated at 11.59% worldwide and 8.1% in the United States [10, 18]. Variations in the prevalence have been noted depending on criterion of symptoms only, TFOS DEWS II criteria, or signs only [18]. In our meta-analysis we focused on the prevalence of symptomatic dry eye to concentrate on outcomes shown to be most important to patients [52]. Dry eye prevalence has also been noted to vary dependent on country income classification level by the World Bank. Studies included in our systematic review reported dry eye prevalence for Mexico, Brazil and Chile; According to 2023 World Bank Classification, Mexico and Brazil are upper-middle income countries, while Chile is a high-income country [53, 54]. Economic status may influence health literacy, access to care, and burden of chronic conditions and it is possible that these factors impact the burden of dry eye among Central and South American populations [55].

### Study limitations

Our initial search for studies was limited to Ovid MEDLINE and Embase which aligned with recommendations by Cochrane [56]. There could be studies beyond these databases, like Scopus and the Latin America and the Caribbean literature on health sciences (LILACS) database, that were not searched in our systematic review. We recognize that our search strategy was conducted two years ago; and we mitigated this by integrating recent literature into the discussion.

## Conclusions

Overall, there is some evidence that dry eye is highly prevalent in young and older South American adults and occupational exposures may have an impact. Low-cost interventions such as awareness campaigns and environmental modifications in university and workplace settings to improve local ergonomics could mitigate development, progression and complications of DED and reduce the evident healthcare burden among eye clinics [57, 58]. We recommend cautious interpretation of these high prevalence estimates due to the enriched populations with respect to risk factor exposures and the risk of bias in the primary studies.

### Supplementary Information

**Supplementary file 1:** **eFigure S1.** PRISMA Search Flow Diagram. **eFigure S2.** **eFigure S3.** Meta-analysis of dry eye prevalence among student and indoor working populations exposed to sustained computer use. **eTable S1.** MEDLINE and Embase search strategies. **eTable S2.** Risk of bias assessments for prevalence studies. **eTable S3.** Stratified associations with dry eye and meibomian gland dysfunction. **eTable S4.** Univariable model associations with dry eye and meibomian gland dysfunction. **eTable S5.** Multivariable model associations with dry eye and meibomian gland dysfunction.

## Data Availability

The datasets used and/or analyzed during the current study are available from the corresponding author on reasonable request.

## Abbreviations

CI: Confidence interval
DED: Dry eye disease
DEWS: Dry Eye Workshop
MGD: Meibomian gland dysfunction
PRISMA: Preferred reporting items for systematic reviews and meta-analyses
TFOS: Tear film and ocular surface society
