# Supplementary material for: Prevalence of dry eye and Meibomian gland dysfunction in Central and South America: a systematic review and meta-analysis
Source: BMC Ophthalmol. 2024 Jan 31;24:50. doi: 10.1186/s12886-023-03249-w (PMC10829227; doi:10.1186/s12886-023-03249-w)
Supplement: Supplementary file 1 — eFigure S1. PRISMA Search Flow Diagram. eFigure S2. eFigure S3. Meta-analysis of dry eye prevalence among student and indoor working populations exposed to sustained computer use. eTable S1. MEDLINE and Embase search strategies. eTable S2. Risk of bias assessments for prevalence studies. eTable S3. Stratified associations with dry eye and meibomian gland dysfunction. eTable S4. Univariable model associations with dry eye and meibomian gland dysfunction. eTable S5. Multivariable model associations with dry eye and meibomian gland dysfunction. [file 12886_2023_3249_MOESM1_ESM.docx]

**Supplemental Online Content for Prevalence of Dry Eye and Meibomian Gland Dysfunction in Central and South America: A Systematic Review and Meta-analysis**

**Table of contents:**

**eFigure 1** PRISMA Search Flow Diagram

**eFigure 2** Forest plot of dry eye prevalence among population-based studies

**eFigure 3** Meta-analysis of dry eye prevalence among student and indoor working populations exposed to sustained computer use

**eTable 1** MEDLINE and Embase search strategies

**eTable 2** Risk of bias assessments for prevalence studies

**eTable 3** Stratified associations with dry eye and meibomian gland dysfunction

**eTable 4** Univariable model associations with dry eye and meibomian gland dysfunction

**eTable 5** Multivariable model associations with dry eye and meibomian gland dysfunction

**Identification of new studies via other methods**

**Identification of new studies via databases and registers**

Records identified from:

Organisations (n = 0)

Practice guidelines (n = 0)

Systematic review citations screening (n = 0)

Citation searching (n = 0)

Records removed *before screening*:

Duplicate records removed

(n = 2612)

Records identified from:

Databases (n = 13745)

- Ovid Medline (n = 5825)
- Embase (n = 7920)

**Identification**

Records excluded

- Irrelevant populations (n = 11317)

Records screened

(n = 11333)

Reports not retrieved

(n = 0)

Reports sought for retrieval

(n = 0)

**Screening**

Reports excluded:

(n = 0)

Reports assessed for eligibility

(n = 0)

Records excluded:

- Ineligible population (n = 1)
- Ineligible outcome (n = 1)

Reports assessed for eligibility

- Total (n = 16)

**Included**

Total studies included in review

(n = 14)

Reports of total included meta-analysis (n = 8)

**eFigure 2** Forest plot of dry eye prevalence among population-based studies

**
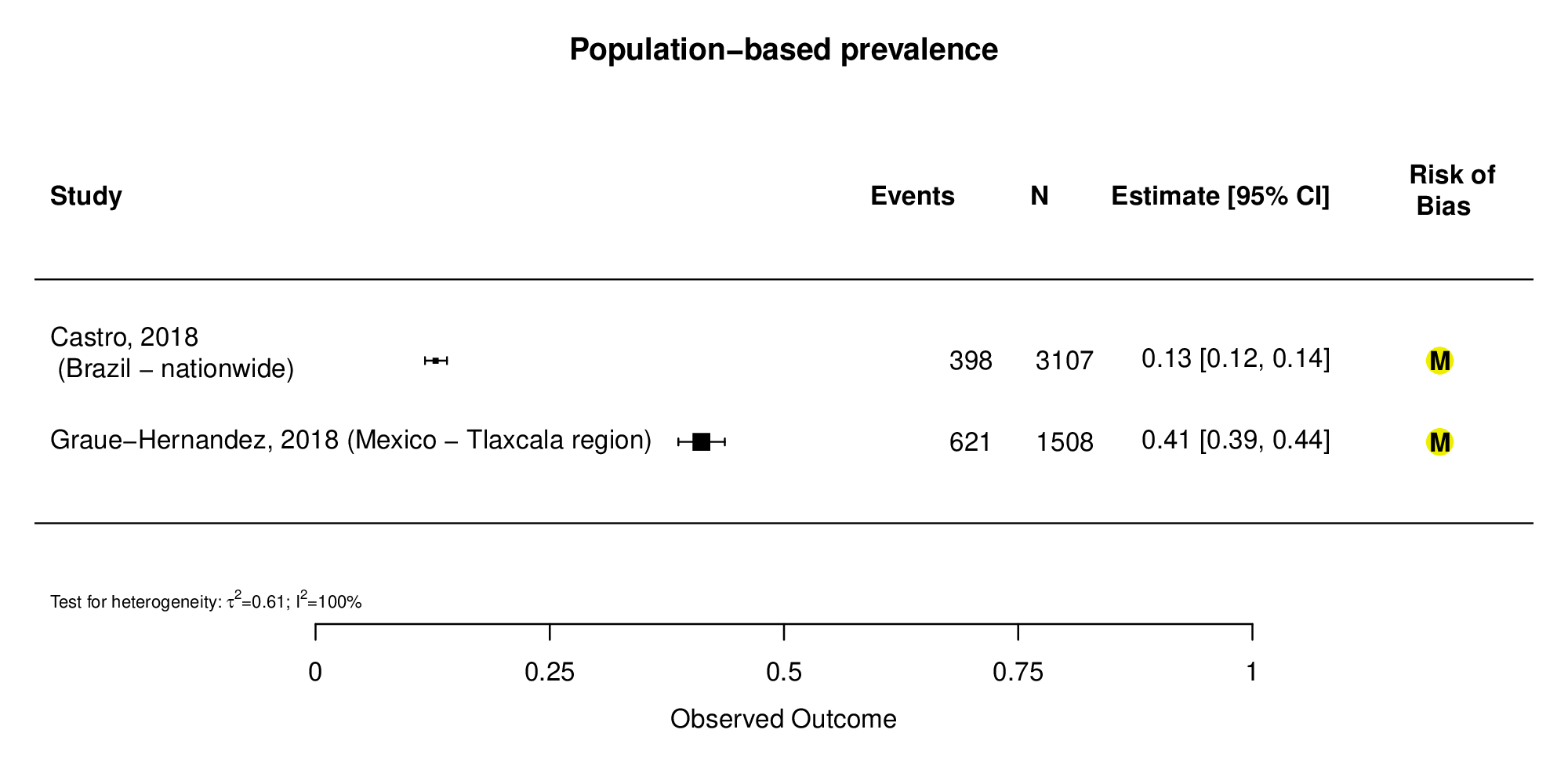
**

**eFigure 3** Meta-analysis of dry eye prevalence among student and indoor working populations exposed to sustained computer use


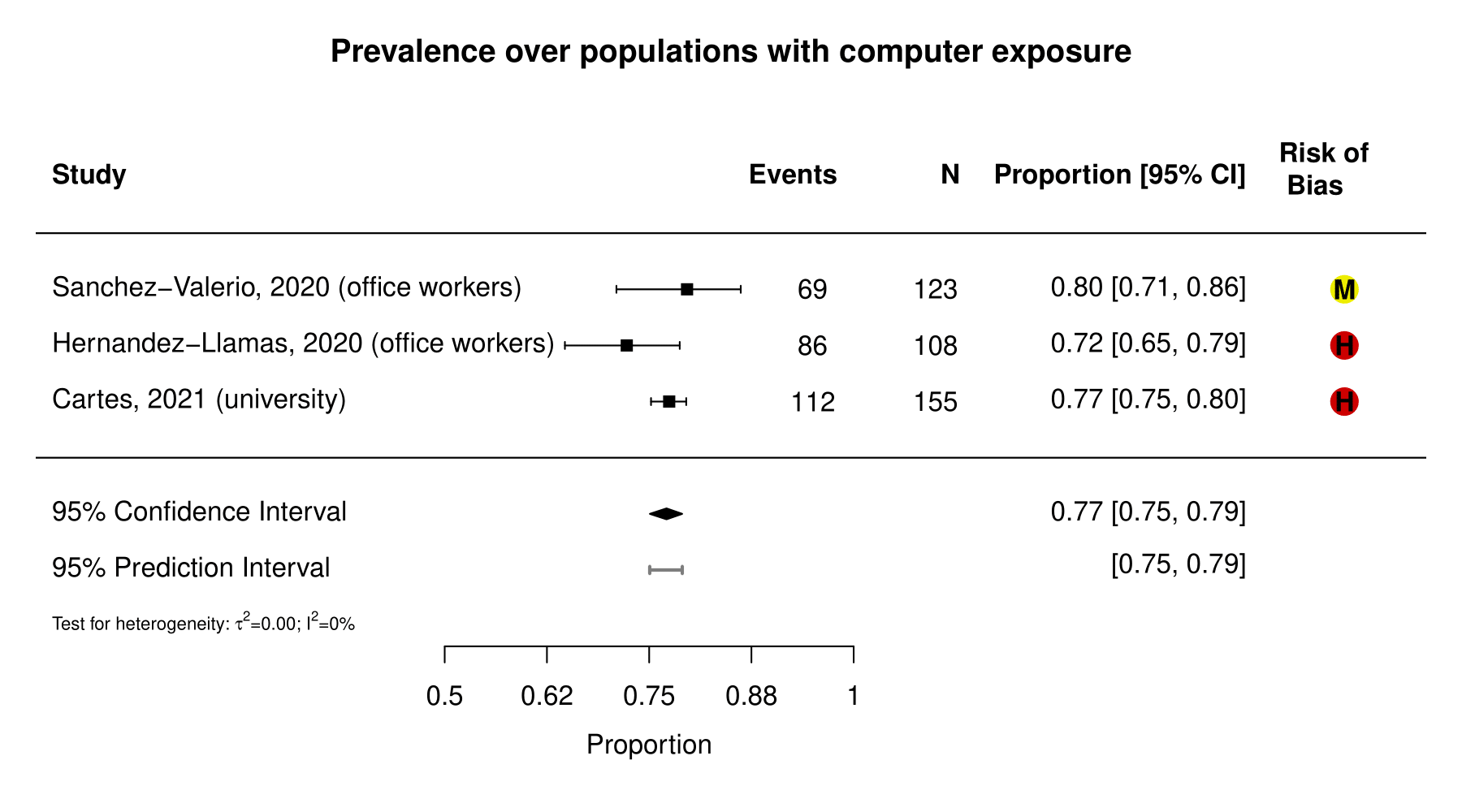


**eTable 1** MEDLINE and Embase search strategies

| **MEDLINE (via Ovid MEDLINE® ALL)**  1 exp Dry Eye Syndromes/  2 exp Keratoconjunctivitis Sicca/  3 exp Xerophthalmia/  4 exp Meibomian Glands/  5 (dry* adj3 eye*).tw,kf.  6 ((keratoconjunctivitis or kerato-conjunctivitis) adj1 sicca).tw,kf.  7 xerophthalmi*.tw,kf.  8 meibomian gland dysfunction.tw,kf.  9 exp Sjogren's Syndrome/  10 ((Sjogren* or Sjoegren*) adj1 (syndrom* or disease*)).tw,kf.  11 (9 or 10) and (exp Eye/ or eye*.mp. or ocular*.mp. or ophthalm*.mp.)  12 or/1-8,11  13 exp Epidemiology/  14 exp Epidemiologic Methods/  15 epidemiology.fs.  16 burden of disease.tw,kf.  17 DALY*.tw,kf.  18 death rate*.tw,kf.  19 Disability Adjusted Life Years.tw,kf.  20 disease burden.tw,kf.  21 endemic*.tw,kf.  22 epidemic*.tw,kf.  23 epidemiolog*.tw,kf.  24 frequency.tw,kf.  25 incidence*.tw,kf.  26 morbidities.tw,kf.  27 morbidity.tw,kf.  28 occurrence.tw,kf.  29 outbreak*.tw,kf.  30 prevalence.tw,kf.  31 surveillance.tw,kf.  32 survival rate*.tw,kf.  33 years lived with disability.tw,kf.  34 years of life lost.tw,kf.  35 YLD*.tw,kf.  36 YLL*.tw,kf.  37 or/13-36  38 12 and 37  39 38 NOT (exp animals/ NOT exp humans/)  40 limit 39 to yr="2010 -Current" |
| --- |
| **Embase (via Elsevier)**  #1 'dry eye'/exp  #2 'dry eye syndrome'/exp  #3 'evaporative dry eye disease'/exp  #4 'keratoconjunctivitis sicca'/exp  #5 'xerophthalmia'/exp  #6 'meibomian gland'/exp  #7 (dry* NEAR/3 eye*):ab,ti,kw  #8 ((keratoconjunctivitis or kerato-conjunctivitis) NEAR/1 sicca):ab,ti,kw  #9 xerophthalmi*:ab,ti,kw  #10 'meibomian gland dysfunction':ab,ti,kw  #11 'Sjoegren syndrome'/exp  #12 ((Sjogren* or Sjoegren*) NEAR/1 (syndrom* or disease*)):ab,ti,kw  #13 (#11 OR #12) AND ('eye'/exp OR eye* OR ocular* OR ophthalm*)  #14 #1 OR #2 OR #3 OR #4 OR #5 OR #6 OR #7 OR #8 OR #9 OR #10 OR #13  #15 'epidemiology'/exp  #16 epidemiology:lnk  #17 'burden of disease':ab,ti,kw  #18 DALY*:ab,ti,kw  #19 'death rate*':ab,ti,kw  #20 'Disability Adjusted Life Years':ab,ti,kw  #21 'disease burden':ab,ti,kw  #22 endemic*:ab,ti,kw  #23 epidemic*:ab,ti,kw  #24 epidemiolog*:ab,ti,kw  #25 frequency:ab,ti,kw  #26 incidence*:ab,ti,kw  #27 morbidities:ab,ti,kw  #28 morbidity:ab,ti,kw  #29 occurrence:ab,ti,kw  #30 outbreak*:ab,ti,kw  #31 prevalence:ab,ti,kw  #32 surveillance:ab,ti,kw  #33 'survival rate*':ab,ti,kw  #34 'years lived with disability':ab,ti,kw  #35 'years of life lost':ab,ti,kw  #36 YLD*:ab,ti,kw  #37 YLL*:ab,ti,kw  #38 #15 OR #16 OR #17 OR #18 OR #19 OR #20 OR #21 OR #22 OR #23 OR #24 OR #25 OR #26 OR #27 OR #28 OR #29 OR #30 OR #31 OR #32 OR #33 OR #34 OR #35 OR #36 OR #37  #39 #14 AND #38  #40 #39 NOT ([animals]/lim NOT [humans]/lim)  #41 #40 AND [2010-2021]/py |

**eTable 2** Risk of bias assessments for prevalence studies

|  |  | General population-based studies | | Working populations | | | Student populations | | | Hospital- and clinic-based populations | | | | | |
| --- | --- | --- | --- | --- | --- | --- | --- | --- | --- | --- | --- | --- | --- | --- | --- |
| **Risk of bias domains** | **Risk of bias items** | Castro, 2018 | Graue-Hernandez, 2018 | Castellanos-Gonzalez, 2016 | Sanchez-Valerio, 2020 | Hernandez-Llamas, 2020 | Garza-Leon, 2016 | Cartes, 2021 | Garza-Leon, 2021 | Skare, 2012 | Martinez JD, 2016 | Garza-Leon, 2017 | da Cruz, 2018 | Surmacz, 2021 | De Freitas, 2021 |
| **External validity** | Representativeness of target population to national population | N | N | N | N | N | N | N | N | N | N | N | N | N | N |
| **Internal validity** | Representativeness of sampling frame to target population | Y | N | Y | N | Y | Y | N | Y | N | Y | N | N | N | N |
| **Sampling bias** | Sampling: random or census | Y | Y | Y | N | Y | Y | N | Y | N | N | Y | Y | N | N |
|  | Minimal non-response bias | Y | N | Y | N | N | Y | N | Y | N | Y | Y | N | N | N |
| **Ascertainment bias** | Data collected directly from participants | Y | Y | Y | Y | Y | Y | Y | Y | Y | Y | Y | Y | Y | Y |
|  | Acceptable case definition | Y | Y | Y | Y | Y | Y | Y | Y | Y | Y | Y | Y | Y | Y |
|  | Valid and reliable instrument | Y | Y | Y | Y | Y | Y | Y | Y | Y | Y | Y | Y | Y | Y |
|  | Same mode of data collection for all participants | Y | Y | Y | Y | Y | Y | Y | Y | Y | Y | Y | Y | Y | Y |
| **Information bias** | Prevalence period appropriate | Y | Y | Y | Y | Y | Y | Y | Y | Y | Y | Y | Y | Y | Y |
|  | Numerator(s) and denominator(s) appropriate | Y | Y | Y | Y | Y | Y | Y | Y | Y | Y | Y | Y | Y | Y |
| **Overall bias** | Overall risk of bias | M | M | M | M | H | M | H | M | H | M | M | H | H | H |

**eTable 3** Stratified associations with dry eye and meibomian gland dysfunction

| Author, Year | Dry eye test used | Sex | Age (years) | Race/  Ethnicity | Severity of dry eye symptoms | Ocular comorbidities | Medical comorbidities | Medication use | Other |
| --- | --- | --- | --- | --- | --- | --- | --- | --- | --- |
| Castellanos-Gonzalez, 2016 | OSDI, TBUT <10s, MGD, Oxford | Males: 52%^1^ Females: 67%^1^ | - | - | OSDI Mild: 21% Moderate: 18% Severe: 17% | (TBUT, Oxford) Chronic eye disease: 58%, 16% Contact lenses: 81%, 44% Refractive surgery: 68%, 14% | (TBUT, Oxford) Allergies: 66%, 16% | (TBUT, Oxford) Hormonal contraceptives: 56%, 13% | (TBUT, Oxford) Residency year 1st: 79%, 33% 2nd: 63%, 17% 3rd: 47%, 15% 4th: 68%, 35% 5th: 32%, 14% 6th: 56%, 11% 7th: 12%, 0% Smoking: 50%, 24% Makeup use: 71%, 12% Microscope use: 68%, 22% |
| Sanchez-Valerio, 2020 | OSDI | - | - | - | - | - | - | - | Computer exposure time  Mild exposure: 65% Moderate exposure: 76% Severe exposure: 92% |
| Castro, 2018 | Previous clinical dry eye diagnosis or presence of severe dry eye symptoms (WHS questionnaire for DES) | (Total dry eye, severe dry eye) Males: 9.8%, 3.2%^1^ Females: 14.4%, 5.8%^1^ | (Total dry eye, severe dry eye) 18-39: 9.9%, 3.1%^1^ 40-60: 13.2%, 4.9%^1^ 60+: 21.1%, 10.3%^1^ | - | - | (Total dry eye, severe dry eye) Ocular surgery: 24.1%, 12.5%^1^ Contact lens use: 19.4%, 4.8%^1^ | (Total dry eye, severe dry eye) Diabetes:  16.6%, 7.3%^1^ Menopause: 23.8%, 11.9%^1^ Rheumatologic diseases: 22.4%, 9.1%^1^ Cancer treatment: 31.4%, 11.4%^1^ | (Total dry eye, severe dry eye) Antidepressants: 24.1%, 9.3%^1^ Anti-allergy: 23.9%, 10.3%^1^ | (Total dry eye, severe dry eye) Smoking:  15.5%, 8.3%^1^ Computer use >6h/day: 15.7%, 5.4%^1^ North region: 11.2%, 3.6%^1^ Northeast region: 18.2%, 15.7%^1^ Central-west region: 12.8%, 9.3%^1^ Southeast region: 11.3%, 8.8%^1^ South region: 17.4%, 15.5%^1^ |
| Cartes, 2021 | DEQ-5 | Males: 63%^1^ Females: 85.4%^1^ | - | - | Mild/moderate: 42.8% Severe: 34.7% | Keratoconus: 87.5%^1^ Allergic conjunctivitis: 92.3%^1^ LASIK: 87.5%^1^ Contact lens use: 91.4%^1^ | Hypertension: 91.6%^1^ Diabetes mellitus: 70%^1^ Thyorid disease: 85.7%^1^ Acne: 81.7%^1^ Rosacea: 84.6%^1^ Rheumatoid arthritis: 66.6%^1^ Systemic Erythematosus: 100%^1^ Depression: 80.7%^1^ Allergies: 16.7%^1^ | Antidepressants: 81.4%^1^ Oral contraceptives: 15.6%^1^ Anti-allergy: 83.6%^1^ Isotretinoin: 73.3%^1^ | Smoking: 79.1%^1^ |
| De Freitas, 2021 | OSDI | - | - | - | Normal: 50%^2^, 60.8%^3^ Mild: 13.3%_2_, 21.7%_3_ Moderate: 16.6%^2^, 8.6%^3^ Severe 20.0%^2^, 8.6%^3^ | - | Diabetes:  38.3%  Menopause: 89.2%^4^ | Metformin: 82.6%^4^ Insulin: 54.3%^4^ | Smoking: 8.6%^4^ |
| Surmacz, 2021 | Schirmer I without anesthesia ≤ 5mm (≥1 eye) | Males: 31.8%^1^ Females: 50.5%^1^ | - | Euro-descent 50%^1^ Afro-descent 25.7%^1^ Asian 50%^1^ | Moderate: 28.1%^4^ Severe: 17.0%^4^ | - | Diabetes mellitus: 50%^1^ Hypertension: 60%^1^ Dyslipedemia: 50%^1^ | - | Ever-smokers: 37.5%^1^ Ex-smokers: 50%^1^ Never-smokers: 44.9%^1^ Categorical BMI Underweight: 33%^1^ Normal: 37%^1^ Overweight: 51%^1^ Obese: 46.2%^1^ |
| Skare, 2012 | Schirmer I without anesthesia ≤ 10mm (≥1 eye) | N/A (100% female cohort) | - | - | - | - | Pregnant: 17.3%^1^ Nonpregnant: 6.6%^1^ | - | - |
| Garza-Leon, 2021 | OSDI | Males: 60.4%^1^ Females: 69.3%^1^ | - | - | Mild: 18.6% Moderate: 15.7% Severe: 31.1% | - | - | - | - |
| Garza-Leon, 2016 | OSDI | Males: 63.7%^1^ Females: 74.8%^1^ | - | - | Mild: 19.9% Moderate: 14.8% Severe 35.7% | - | - | - | - |
| Garza-Leon, 2017 | OSDI | Males: 84.2%^1^ Females: 88.4%^1^ | 18-30: 83.5% 31-40: 82.9% 41-50: 86.5% 51-60: 89.3% 61-70: 86.9% 71-80: 89.7% 81-90: 93.4% 91-100: 100% | - | Mild: 13.6% Moderate: 13.4% Severe: 59.6% | - | - | - | - |
| Graue-Hernandez, 2018 | DEQ-5 | Males: 35.0%^1^ Females: 45.3%^1^ | - | - | Mild/Moderate: 30.2% Severe: 11.0% | Normal visual acuity: 38.9%^1^ Moderate visual impairment: 59.4%^1^ Severe visual impairment/ blindness: 68.6%^1^ Cataract surgery: 45.2%^1^ Wears glasses: 45.2%^1^ | Diabetes mellitus: 43.1%^1^ Hypertension: 44%^1^ | Antihypertensive: 49.4%^1^ Hypoglycemic: 42.9%^1^ | Smoking ≥ 10 pack years: 58.4%^1^ Smoking < 10 pack years: 46.3%^1^ Nonsmokers: 36.1%^1^ Current alcohol use: 42.6%^1^ Former alcohol use: 45.0%^1^ Never alcohol use: 35.1%^1^ Less than elementary school education: 41.5%^1^ Elementary school or higher: 41.1%^1^ Rural: 42.8%^1^ Urban: 40.7%^1^ |
| Martinez-JD, 2016 | OSDI, DEQ-5, Schirmer's ≤5, TBUT, MGD, corneal staining | Severe DES per OSDI Female: 44%^1^ Male: 42%^1^ | DES per DEQ-5 (not reported for all age ranges) 46-55: 36% 66-75: 18% 76-85: 38% | - | OSDI Mild/moderate: 35% Severe: 43% DEQ-5 Mild: 44% Severe: 30% | Severe DES per OSDI Contact lens use: 27.2%^1^ Previous surgery: 51.7%^1^ | Severe DES per OSDI Diabetes: 45%^1^ Arthritis: 43.8%^1^ Thyroid problems: 75%^1^ Dry mouth: 65.5%^1^ Acne: 55%^1^ Depression: 60%^1^ | Severe DES per OSDI Eyedrops: 48%^1^ Antihypertensive: 54.7%^1^ Antihistamine: 55.6%^1^ Diuretics: 33.3%^1^ GI ulcer medication: 62.1%^1^ Multivitamins: 42.9%^1^ | Severe DES per OSDI indoor occupation: 37.3%^1^ Current smoker: 45.5%^1^ Air conditioning exposure: 48.3%^1^ |
| Hernandez-Llamas, 2020 | OSDI | - | - | - | Construction workers Mild: 13.4% Moderate: 9.4% Severe: 12.8% Office workers: Mild: 18.1% Moderate: 13.5% Severe: 40.6% | - | - | - | - |
| n.r.: not reported | | | | | | | | | |
| 1 Among all with the characteristic | | | | | | | | | |
| 2 Among normal controls | | | | | | | | | |
| 3 Among diabetes cases | | | | | | | | | |
| 4 among dry eye cases | | | | | | | | | |

**eTable 4** Univariable model associations with dry eye and meibomian gland dysfunction

| Author, Year | Dry eye test used | Sex (OR [95% CI]) | Age (OR [95% CI]) | Ocular comorbidities (OR [95% CI]) | Medical comorbidities (OR [95% CI]) | Medication use (OR [95% CI]) | Other (OR [95% CI]) |
| --- | --- | --- | --- | --- | --- | --- | --- |
| Sanchez-Valerio, 2020 | OSDI >12 | - | - | - | - | - | Computer exposure time TBUT (Spearman's rank correlation test, p<= 0.05) cumulative hours/total years of work: Rho -0.376, p <0.000 Hours/day: Rho -0.467, p <0.000 Days/week: Rho -0.134, p 0.167 Hours/week: Rho -0.463, p <0.000 Hours/year: Rho -0.463, p <0.000 |
| Castro, 2018 | Previous clinical dry eye diagnosis or presence of severe dry eye symptoms (WHS questionnaire for DES) | Female: 1.49 [1.15- 1.92]^1^ | ≥ 40y: 1.47 [1.16-1.87] ≥60y: 2.02 [1.53-2.66] | Ocular Surgery: 2.31 [1.70-3.13] Contact Lens use: 1.82 [1.31-2.53] | Diabetes 1.45 [0.99-2.13] Menopause: 1.92 [1.37-2.68] Connective Tissue Disorder 1.93 [1.23-3.02] Cancer Treatment: 3.59 [1.71-7.55] | Antidepressants:  2.21 [1.57-3.09] Anti-allergy: 2.33 [1.73-3.14] | Smoking: 1.44 [0.83-2.48] Computer Use >6h: 1.49 [1.16-1.92] |
| Cartes, 2021 | DEQ-5 >6 | Female: 2.57 [1.97–3.35]^1^ | - | Keratoconus: 5.56 [1.27–24.44] Contact lens use: 1.77 [1.24–2.53] | Depression: 1.57 [0.99–2.48] Allergies:  1.63 [1.24–2.13] | Isotretinoin: 2.10 [0.86–4.99] | Smoking: 1.32 [0.94–1.84] Screen exposure (hrs) 1.02 [1.01–1.05] |
| Surmacz, 2021 | Schirmer I without anesthesia ≤ 10mm (≥1 eye) | Female 2.1 [1.02-4.6]^1^ | 1.04 [1.01-1.06] | - | - | - | - |
| Garza-Leon, 2021 | OSDI >22 | Female 1.84 [1.37-2.46] | - | Contact lens use: 2.76 [1.44-5.26] Contact lens type: RGP 0.69 [0.15-3.09]^2^ Contact lens use overnight: 1.11 [0.24-5.16] | - | - | - |
| Garza-Leon, 2016 | OSDI | Female: 1.29 [1.13-1.48] | - | Refractive surgery: 0.82 [0.61, 1.09] Contact lens use: 1.12 [0.84-1.48] | - | Eyedrop use: 2.00 [1.65-2.4] | Smoking: 1.24 [1.06-1.46] Hrs in front of computer: 0.82 [0.72-0.93] |
| Garza-Leon, 2017 | OSDI | Female:  1.17 [1.08-1.28] | 1.32 [1.21-1.43] | - | - | - | Physician specialty^3^ Ocular inflammatory diseases: 0.44 [0.34-0.57] Retina: 0.82 [0.67-0.98] Cornea: 1.01 [0.90-1.14] Glaucoma: 1.05 [0.90-1.23] |
| Martinez-JD, 2016 | OSDI, DEQ-5, Schirmer's ≤5, TBUT, MGD, corneal staining | MGD Male: 1.7 [1.04-2.6] | MGD 1.07 [1.05-1.09] | Contact lens use: MGD 0.01 [0.02-0.5] | Dry mouth:  OSDI 3.0 [1.6-5.5] ATD 1.9 [1.02-3.6] MGD Arthritis: 7.7 [1.001-59] | OSDI GI ulcer medication: 2.3 [1.1-5.1] ATD  Diuretic use: 7.4 [1.3–41] MGD Antihypertensive 2.7 [1.3-5.7] | - |
| Hernandez-Llamas, 2020 | OSDI | Female: 3.82 [2.48-5.91] | - | Contact lens use: 4.67 [1.88-11.57] Ocular disease significant OR>1 but specific OR and CI not reported | Systemic disease significant OR>1 but specific OR and CI not reported | - | Office workers 4.15 [2.52-6.85]^4^ |
| 1 Reference: Male | | | | | | | |
| 2 Reference: soft contact lens | | | | | | | |
| 3 Reference: anterior segment | | | | | | | |
| 4 Reference: construction workers | | | | | | | |
| MGD: International Workshop on Meibomian Gland Dysfunction (2011) | | | | | | | |

**eTable 5** Multivariable model associations with dry eye and meibomian gland dysfunction

| Author, Year | Dry eye test used | Sex (OR [95% CI]) | Age (OR [95% CI]) | Ocular comorbidities (OR [95% CI]) | Medical comorbidities (OR [95% CI]) | Medication use (OR [95% CI]) | Other (OR [95% CI]) |
| --- | --- | --- | --- | --- | --- | --- | --- |
| Castro, 2018 | Previous clinical dry eye diagnosis or presence of severe dry eye symptoms (WHS questionnaire for DES) | Female:  1.47 [1.12-1.93] | ≥60y: 2.00 [1.44-2.77] | Ocular Surgery: 1.84 [1.30-2.60] Contact Lens use: 1.93 [1.36-2.73] | Cancer treatment: 3.03 [1.39-6.59] | Antidepressants: 1.61 [1.12-2.31] Anti-allergy: 2.11 [1.54-2.89] | Computer use >6h: 1.77 [1.36-2.31] |
| Cartes, 2021 | VDT-related symptoms aggregate score | Female: 3.38 [2.72–4.04] | - | - | - | Anti-allergy: 1.96 [1.15–2.79] | Screen exposure (hrs): 0.15 [0.09–0.20] |
| Garza-Leon, 2021 | OSDI >22 | Female 4.71[1.10-20.17] | - | Contact lens use: 2.61 [1.36-5.01] Contact lens type: RGP 0.90 [0.34-2.443]^2^ Contact lens use overnight: 0.98 [0.19-5.07] | - | - | - |
| Graue-Hernandez, 2018 | DEQ-5 | Female: 2.26 [1.70-3.00] | - | - | - | Antihypertensive: 1.29 [1.00-1.65] | Smoking ≥ 10 pack years: 2.29 [1.44-3.63] Smoking < 10 pack years: 1.40 [1.05-1.87] Ever alcohol use: 1.31 [1.02-1.70] |
| Martinez-JD, 2016 | OSDI, DEQ-5, Schirmer's ≤5, TBUT, MGD, corneal staining | - | Older age significant association with MGD [n.r.] | Contact lens use significant association with lower MGD [n.r.] | Dry mouth significant association with severe DE symptoms per OSDI [n.r.] | GI ulcer medication significant association with severe DE symptoms per OSDI [n.r.] | - |
| Hernandez-Llamas, 2020 | OSDI | - | - | Ocular disease significant OR>1 but specific OR and CI not reported | Systemic disease significant OR>1 but specific OR and CI not reported | - | - |
| 1 Reference: Male | | | | | | | |
| 2 Reference: soft contact lens | | | | | | | |
